# Proteomic and cellular localisation studies suggest non‐tight junction cytoplasmic and nuclear roles for occludin in astrocytes

**DOI:** 10.1111/ejn.13933

**Published:** 2018-05-30

**Authors:** Sarah V. Morgan, Claire J. Garwood, Luke Jennings, Julie E. Simpson, Lydia M. Castelli, Paul R. Heath, Simeon R. Mihaylov, Irina Vaquéz‐Villaseñor, Thomas C. Minshull, Paul G. Ince, Mark J. Dickman, Guillaume M. Hautbergue, Stephen B. Wharton

**Affiliations:** ^1^ Sheffield Institute for Translational Neuroscience University of Sheffield Sheffield UK; ^2^ Department of Chemical and Biological Engineering University of Sheffield Sheffield UK

**Keywords:** astrocytes, RNA metabolism, tight junction proteins

## Abstract

Occludin is a component of tight junctions, which are essential structural components of the blood–brain barrier. However, occludin is expressed in cells without tight junctions, implying additional functions. We determined the expression and localisation of occludin in astrocytes in cell culture and in human brain tissue, and sought novel binding partners using a proteomic approach. Expression was investigated by immunocytochemistry and immunoblotting in the 1321N1 astrocytoma cell line and ScienCell human primary astrocytes, and by immunohistochemistry in human autopsy brain tissue. Recombinant N‐ and C‐terminal occludin was used to pull‐down proteins from 1321N1 cell lysates and protein‐binding partners identified by mass spectrometry analysis. Occludin was expressed in both the cytoplasm and nucleus of astrocytes in vitro and in vivo. Mass spectrometry identified binding to nuclear and cytoplasmic proteins, particularly those related to RNA metabolism and nuclear function. Occludin is expressed in several subcellular compartments of brain cell‐types that do not form tight junctions and the expression patterns in cell culture reflect those in human brain tissue, indicating they are suitable model systems. Proteomic analysis suggests that occludin has novel functions in neuroepithelial cells that are unrelated to tight junction formation. Further research will establish the roles of these functions in both cellular physiology and in disease states.

**Abbreviations:**
BBB: Blood brain barrier
DDX3X: Deadbox helicase 3
eEf2: Eukaryotic translation elongation factor
FACT: Facilitates chromatin transcription
HSPA14: Heat shock protein family A 14
MAGUK: Membrane associated guanylate kinase
MCI: Mild cognitive impairment
MDCK: Madin‐Darby canine kidney cells
MS: Mass spectrometry
O/N: Overnight
RNP: Ribonucleoprotein
RT: Room temperature
SSRP1: Structure specific recognition protein 1
STRING: search tool for the retrieval of interacting genes/proteins
ZO: Zona‐occludens

## 1 INTRODUCTION

Tight junctions formed by brain endothelial cells are a key component of the blood–brain barrier (BBB), limiting paracellular and intramembranous diffusion and giving the cell polarity (Chow & Gu, 2015; Zlokovic, 2008). Changes in the expression of tight junctions have been associated with leakage of the BBB in various acute and chronic neurological disorders (Dallasta et al., 1999; Kirk, Plumb, Mirakhur, & McQuaid, 2003; Nag, Kapadia, & Stewart, 2011; Popescu et al., 2009; Sandoval & Witt, 2008).

Tight junctions are composed of a multi‐molecular complex of proteins that can be divided into integral membrane proteins and cellular scaffolding proteins (Guillemot, Paschoud, Pulimeno, Foglia, & Citi, 2008; Haseloff, Dithmer, Winkler, Wolburg, & Blasig, 2015). Occludin is a tight junction‐associated marvel protein (TAMP), characterised by four transmembrane MARVEL (myelin and lymphocyte [MAL] and related proteins for vesicle trafficking and membrane link) domains that target it to the cell membrane (Yaffe et al., 2012). Occludin binds zonula occludens 1 (ZO‐1), a member of the membrane‐associated guanylate kinase (MAGUK)‐like proteins through the hairpin structure formed by the SH3‐U5‐GUK‐U6 region, thus establishing a link between occludin and the actin cytoskeleton (Fanning & Anderson, 2009; Fanning et al., 2007; Guillemot et al., 2008). Basic structural information for occludin is illustrated in Figure S1.

Although the protein components of tight junctions are well characterised, our understanding of the molecular structure and protein interactions of tight junctions is more limited. Claudins are another tight junction protein, and to date 24 claudins have been identified in humans. Gene manipulation studies which selectively knockout either occludin or claudin(s) have demonstrated quite differing effects on tight junction function/integrity. Occludin knockout mice have a complex phenotype, with considerable growth retardation and alterations in their sexual behaviour but they exhibit no morphological abnormalities in their tight junctions and barrier function appears normal (Saitou et al., 2000; Schulzke et al., 2005). This contrasts with claudin‐deficient mice in which barrier function is disturbed; for example claudin‐1 null mice do not survive post‐natally due to a compromised epidermal barrier (Furuse et al., 2002) while claudin‐5 deficient mice have a “leaky” BBB which also results in death post‐natally (Nitta et al., 2003). These findings, along with the observation that occludin is expressed by neuroepithelial cells that do not possess tight junctions, suggest that the function of occludin is more complex than previously supposed.

In studies of BBB alterations with Alzheimer‐type pathology in an ageing human brain cohort, we observed expression of occludin and ZO‐1, but not claudin‐V in glial cells in white matter and cerebral cortex (Simpson et al., 2010; Viggars et al., 2011). We have also shown astrocytic expression of genes for occludin, ZO‐1 and claudins in a gene expression microarray study, with down‐regulation of the tight junction Kyoto encyclopedia of genes and genomes (KEGG) pathway (hsa04530), in addition to other junctional pathways with increasing Braak neurofibrillary tangle stage (Simpson et al., 2011). Several other studies have also documented expression of certain tight junction proteins in cultured astrocytes and in human tissue (Bauer et al., 1999; Howarth, Hughes, & Stevenson, 1992; Romanitan, Popescu, Winblad, Bajenaru, & Bogdanovic, 2007). These data suggest that occludin has functions in neuroepithelial cells other than its role in tight junctions, and that these functions may be perturbed in ageing and neurodegeneration.

In this study we demonstrate that occludin is expressed in human astrocytes in vitro and in vivo, and is localised to the cytoplasm and nucleus, but not to the cell membrane. To investigate possible additional functions, we used mass spectrometry (MS) to identify candidate binding partners of occludin in neuroepithelial cells.

## 2 METHODS

### 2.1 Cell culture

The 1321N1 human astrocytoma cell line was cultured in Dulbecco's Modified Eagle Medium (DMEM) (Gibco), supplemented with 10% foetal bovine serum (FBS) (Sigma) and 1% penicillin/streptomycin (P/S). Human foetal primary astrocytes isolated from the cerebral cortex were purchased from ScienCell Research Laboratories (Carlsbad, CA, USA) and were cultured in ScienCell medium supplemented with FBS, P/S and Astrocyte Growth Supplement (all ScienCell Research Laboratories). The endothelial cell line, hCMEC/D3 (Cedarlane, USA) was cultured in EBM‐2 medium supplemented with vascular endothelial growth factor, insulin‐like growth factor‐1, 0.025% human epidermal growth factor, 0.1% human basic fibroblast growth factor, 0.04% hydrocortisone, 0.1% ascorbic acid and 2.5% FBS (Lonza) on Collagen‐type 1 (Sigma, UK) coated plates.

### 2.2 Western blotting

Once cells had reached approximately 80% confluency they were pelleted and snap frozen in liquid nitrogen. Cell lysates were prepared by resuspending in extra strong lysis buffer (100 mM Tris‐HCl pH 7.5, 75 mM NaCl, 0.5% (w/v) SDS, 20 mM sodium deoxycholate, 1% (v/v) Triton X‐100, 2 mM Na_3_VO_4_, 1.25 mM NaF, 10 mM EDTA, Protease Inhibitor Cocktail (PIC) and PhosSTOP (both Roche, Basel Switzerland). Lysates were then sonicated followed by centrifugation at 17,000 *g*_(av)_ for 30  min at 4°C. The protein concentration of whole cell lysates was measured using a bichionic acid (BCA) protein assay kit (ThermoFisher Scientific, Waltham, MA, USA) and samples were standardised to equal protein concentration before being analysed by SDS‐PAGE. Samples containing 20–30 μg of protein were separated on 10% SDS‐PAGE gels and electrophorectically transferred onto 0.2 μm Hybond‐C extra nitrocellulose membranes (Amersham Laboratories). Membranes were blocked in 5% (w/v) bovine serum albumin (BSA) diluted in 0.05% Tween 20 in PBS for 30 min at room temperature (RT). Membranes were subsequently incubated with an occludin primary antibody overnight (O/N) at 4°C, (71–1,500, 1:200 dilution; ThermoFisher Scientific), α‐tubulin (ABM40035, 1:5,000; Abbkine) was used a loading control. Membranes were then incubated with HRP‐conjugated secondary antibodies (1:5,000 dilution; DAKO) followed by ECL (EZ‐ECL Biological Industries). Detected proteins were visualised using a G:Box Chemi‐XT CCD Gel imaging system (Syngene).

### 2.3 Cell fractionation

1321N1 cells were also fractionated to yield cytoplasmic and nuclear fractions to confirm the subcellular localisation of occludin. For the cytoplasmic fraction, cells were trypsinised, collected in PBS and pelleted by centrifugation at 800 *g*_(av)_ for 5 min. Cell pellets were quickly washed with hypotonic lysis buffer (10 mM HEPES pH 7.9, 1.5 mM MgCl_2_, 10 mM KCl, 0.5 mM DTT) and lysed in hypotonic lysis buffer with 2 mM PMSF (Sigma) and SIGMAFAST PIC tablets, EDTA free (Sigma). Lysates were then subjected to differential centrifugation at 4°C (1,500 *g*_(av)_ for 3 min, then 3,500 *g*_(av)_ for 8 min, and then 17,000 *g*_(av)_ for 1 min), supernatants were transferred to fresh tubes after each centrifugation. Nuclear pellets obtained after centrifugation at 1,500 *g*_(av)_ for 3 min were lysed in Reporter lysis buffer (Promega) for 10 min on ice before centrifugation at 17,000 *g*_(av)_ for 5 min at 4°C. Total fractions were collected in Reporter lysis buffer containing 2‐mM PMSF (Sigma) and PIC prior to lysis for 10 min on ice before centrifugation at 17,000 *g*, 5 min, 4°C. Equal volumes of total, nuclear and cytoplasmic lysates were subjected to western blotting. Membranes were probed with antibodies directed against both the C‐terminal (ThermoFisher Scientific, 71–1,500, 1:200 dilution) and the N‐terminal of occludin (ab167161, 1:10,000 dilution; Abcam) O/N at 4°C. Fractionation was confirmed by western blotting for heat shock protein family A 14 (HSPA14) (ab108612, 1:2,000 dilution; HSPA14 Rabbit Monoclonal; Abcam) which is expressed in the cytoplasm where it functions as a component of the ribosome‐associated complex (Wada, Hamada, & Satoh, 2006) and structure specific recognition protein 1 (SSRP1) (609702, 1:1,000; Biolegend), a nuclear protein which is a component of the facilitates chromatin transcription (FACT) complex (Belotserkovskaya, Saunders, Lis, & Reinberg, 2004).

### 2.4 Immunocytochemistry

Cultured cells were fixed in 4% paraformaldehyde for 10 min at 37°C and then permeabilised in 0.3% Triton X‐100 in PBS (3 min at RT). After further washing, cells were incubated in 3% bovine serum albumin prior to incubating in occludin antibody for 2 hrs at RT (71–1,500, 1:50; Thermo Fisher Scientific). Cells were subsequently washed in PBS and then incubated with the appropriate species of secondary antibody for 2 hrs at RT (1:500 Alexa Fluor conjugated, Life Technologies). Cell nuclei were stained with Hoescht 33342 (5 μg/ml bisbenzimide in PBS). Coverslips were mounted on to glass slides using fluorescent mounting medium (DAKO) and cells were examined using a Nikon DS‐Ri1 Eclipse microscope (Nikon, Kingston, UK).

### 2.5 Immunohistochemistry

Samples of post‐mortem human temporal cortex from cases with Alzheimer's disease, mild cognitive impairment (MCI) and controls were obtained from the Newcastle Brain Tissue Resource (Newcastle and North Tyneside REC 1: 08/H0906/136), following approval by their review committee. Case details are shown in Tables S1 and S2. Immunohistochemistry was performed on formalin fixed paraffin embedded tissue sectioned at 9 μM using a standard avidin biotinylated enzyme complex (ABC) method, and the signal visualised with diaminobenzidine (Vector Laboratories, Peterborough, UK). Antigen retrieval was performed by microwaving for 15 min in EDTA at pH 8.0 and the occludin antibody was used at a concentration of 1:100 O/N at 4°C (Rabbit Polyclonal, 71–1,500, ThermoFisher Scientific). Negative and isotype controls were included to confirm antibody specificity. For dual staining experiments, we used a method we have previously described (Mathur et al., 2015). Briefly, stained sections were subsequently blocked with 1.5 % normal serum, followed by an avidin‐biotin block (Vector Laboratories) and then incubated with glial fibrillary acidic protein (GFAP) primary antibody diluted 1:500 for 1 hr at RT (Rabbit polyclonal, Z0334; DAKO). After incubating in biotinylated secondary antibody (Vectastain Elite kit, Vector Laboratories), the signal was visualised with alkaline phosphatase‐conjugated avidin–biotin complex using the substrate Vector Red (Vector Laboratories). All sections were counterstained with Harris's Haematoxylin before dehydrating, clearing in xylene and mounting with DPX (Sigma) mounting media. Images were captured using a Nikon DS Ri1 Eclipse.

### 2.6 Generation of recombinant occludin

pCMV‐SPORT6 (4,396 bp) containing an occludin (OCLN) cDNA IMAGE clone (Source Bioscience) was hosted in the DH10B *Escherichia coli* (*E. coli*) strain with ampicillin resistance and NotI/EcoRV cloning sites. Primers (Sigma) were designed to amplify the N‐ (OCLN_N 1‐56) and the C‐terminal (OCLN_C 271‐522) domains as well as the full length occludin sequence (OCLN 1‐522) (Table 1) and occludin sequences amplified by touchdown PCR. Products were run on a 2% agarose gel, excised and digested with *Nde*1 and *Xho*1, then ligated into the pET24b‐GB1‐6His vector followed by transformation into the DH5α *E. coli* strain. The DNA sequences of all three inserts were verified by Sanger sequencing (conducted by Source Bioscience). Plasmids containing OCLN_N 1‐65, OCLN_C 271‐522 and OCLN 1‐522 DNA sequences were transformed into the BL21‐RP *E. coli* strain.

**Table 1:** Sequences for occludin primers

Primer	Sequence
OCLN_1_Nde15	5′‐GGCGGGCATATGTCATCCAGGCCTCTTGAAAG
OCLN_271_Nde15	5′‐GGCGGGCATATGGACAGGTATGACAAGTCC
OCLN_522_Xho13	5′‐CCCGCCCTCGAGCTATGTTTTCTGTCTATCATAG
OCLN_65_Xho14	5′‐CCCGCCCTCGAGTTAAATCACTCCTGGAGGAGAGGTC

Expressed proteins were purified from bacterial cultures and analysed by SDS‐PAGE. Briefly, induced cultures were centrifuged, lysed (50 mM Tris, 1M NaCl, 0.5% (v/v) Triton‐X 100, PIC, pH 8.0), sonicated and then centrifuged at 17,000 *g*_(av)_ (7 min, 4°C). The resulting supernatant was added to TALON Metal Affinity Cobalt Beads (Clontech Laboratories) and incubated for 1 hr on a rotary wheel at 4°C. GB1‐tagged protein was then eluted from the beads and western blots utilising antibodies with epitopes located in either the N‐ or C‐terminal domain of occludin were used to confirm the size and successful expression of GB1‐OCLN_ N and GB1‐OCLN_C recombinant proteins (Table 2 for antibodies).

**Table 2:** Antibodies used to confirm the size and successful expression of GB1‐OCLN_ N and GB1‐OCLN_C

Epitope location	Antibody	Species	Dilution	Supplier	Code
N‐terminal	Occludin	Rabbit	1:10,000	Abcam	Ab167161
C‐terminal	Occludin	Rabbit	1:500	ThermoFisher Scientific	71–1,500

### 2.7 Pull‐down protein‐binding assay

Prior to lysis, 1321N1 cells were treated with 5 mM CaCl_2_ and 4 μM A23187 calcium ionophore (Sigma) and incubated at 37°C for 15 min. Cells were lysed in lysis buffer (150 mM NaCl, 25 mM TrisHCl, 1% (v/v) Triton X‐100, 0.05% (w/v) SDS, 10 mM NaF, 1 mM NaVO_4_, PIC [Roche], pH 8.0) and incubated on ice for 30 min, then passed through a 25 G needle 10× and centrifuged at 17,000 *g*_(av)_ for 5 min, after which the supernatants were combined and the concentration determined by Bradford assay. Cell lysates were diluted to 1 mg/ml for pull‐down assays.

GB1‐OCLN_N or GB1‐OCLN_C was adsorbed onto 30 μl IgG bead slurry (GE Healthcare). A GB1‐6His control was also included in each experiment. Beads were then washed and 1321N1 cell lysates were added to the beads with a small quantity retained for use as an input sample. The bead–lysate mixture was incubated O/N at 4°C on a rotary wheel. Tubes were then centrifuged at 0.5 *g*_(av)_ for 2 min and the supernatant discarded. To elute proteins, beads were washed and then boiled at 95°C for 5 min with 2× Laemmli buffer. Eluates were analysed by SDS‐PAGE, protein bands were visualised with either Instant *Blue* coomassie (Expedeon) or a SilverXpress^®^ Silver Staining Kit (Thermo Fisher Scientific), according to the manufacturer's instructions. Bands from two gels representing the GB‐1 OCLN_N and GB1 OCLN_C pull‐down assays were prepared for mass spectrometry as previously described (Pandey, Andersen, & Mann, 2000).

### 2.8 Mass spectrometry – ESI‐MS analysis

Mass spectrometry was performed with an AmaZon ion trap mass spectrometer (Bruker Daltonics). Mass spectra were acquired with automated precursor ion selection. Datasets were converted to Mascot Generic Files using scripts supplied by Bruker and searched against the Swiss‐Prot database (release Jan 2016, 550,116 sequences) with Mascot server 2.5.1 (Matrix Science, London, UK). Search parameters were as follows: enzyme; trypsin; fixed modification carbamiodomethyl (C) and variable modification oxidation (M); maximum missed cleavages = 1. A peptide ion score of ≤15 as a cut‐off as calculated by Mascot was also used to filter. Proteins were identified using a significance threshold *P* < 0.05. Protein identifications were based on a minimum of two unique peptides and a Mascot score ≥ 50 were considered to be significant. The false discovery rates were determined using the Mascot decoy database option and were typically ≤3%.

## 3 RESULTS

### 3.1 Occludin is expressed in human astrocytes

**Figure 1:**
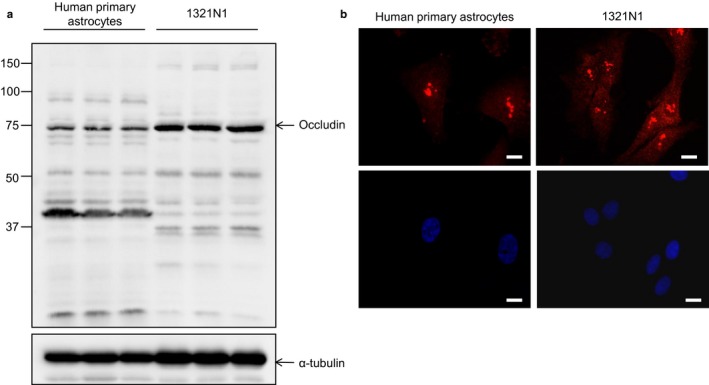
Occludin expression in human primary astrocytes and the human astrocytoma line 1321N1. (a) Western blotting confirms expression of the protein at the expected size as well as additional bands (see main text for description), molecular weight markers are indicated in kDa. (b) Immunocytochemistry for occludin (red) demonstrates both cytoplasmic and nuclear expression of the protein. Nuclei are shown in blue (Hoescht), scale bar represents 10μm [Colour figure can be viewed at http://wileyonlinelibrary.com]

Western blotting of human primary astrocytes and 1321N1 cell lysates confirmed expression of occludin in these cells, with a clear band at 75 kDa as well as additional fainter bands at a number of other molecular weights (Figure 1a). The calculated molecular weight of occludin is 59 kDa; however the supplier states that this antibody recognises a range of higher molecular weight species ranging from 65 to 82 kDa due to hyperphosphorylation of occludin, as well as recognising isoforms at 53 and 23 kDa. Both human primary astrocytes and 1321N1 cells showed a similar banding pattern except for the higher molecular weight bands (human primary astrocytes ~90 kDa, 1321N1 cells ~150 kDa) and the lower molecular weight bands (human primary astrocytes ~40 kDa, 1321N1 cells ~37 kDa).

To confirm that the antibody was specifically detecting occludin we pre‐adsorbed the occludin antibody (Thermofisher 71‐1,500) overnight with recombinant human C‐terminal occludin (aa 271‐522). Pre‐adsorbing the antibody decreased the intensity of the 75 kDa, 50 kDa and the 37 kDa occludin bands confirming the specificity of the antibody (Figure S2).

**Figure 2:**
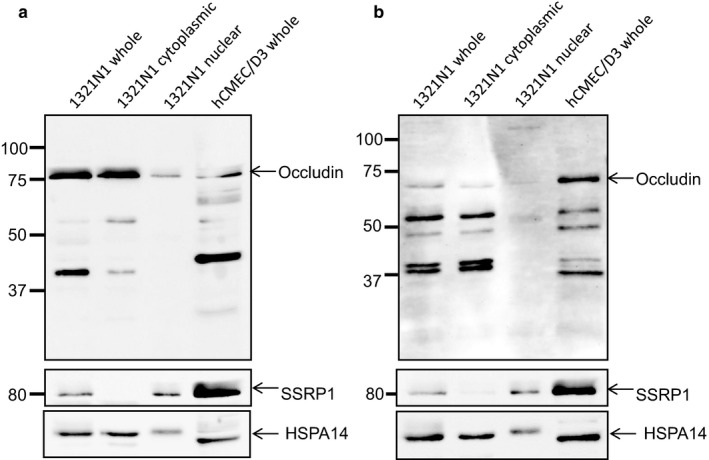
Occludin expression in fractionated human 1321N cells. Western blotting using an antibody directed against the C‐terminal of occludin (a) and the N‐terminal of occludin (b) confirms expression at the expected molecular weights. Whole cell lysates from the endothelial cell line hCMEC/D3 were included as a positive control. Cell fractionation was confirmed using an antibody directed against SSRP1 (nucleus) and HSPA14 (cytoplasm). Molecular weight markers are indicated in kDa

To investigate localisation of occludin we initially used immunocytochemistry, which demonstrated diffuse cytoplasmic expression and punctate nuclear staining for occludin in both human primary astrocytes and the 1321N1 human astrocytoma cell line. This was not accompanied by membrane staining (Figure 1b). To further explore occludin localisation, 1321N1 cells were fractionated into cytoplasmic and nuclear fractions for western blot analysis (Figure 2). Antibodies directed against both the C‐terminal and N‐terminal were used to confirm that cytoplasmic and nuclear occludin possessed the C‐ and N‐terminal of occludin. Whole cell lysates from an endothelial cell line (hCMEC/D3) were included as a positive control. Fractionation was confirmed by probing for proteins exclusively localised with the nucleus (SSRP1) and the cytoplasm (HSPA14). A strong SSRP1 band was detected in the whole cell lysate and nuclear fractions, while HSPA14 was almost exclusively confined to the whole cell lysate and the cytoplasmic fraction. Occludin was present in both the nuclear and cytoplasmic fractions with multiple bands detected with both antibodies. Both antibodies detected bands at the expected predicted molecular weight(s) of occludin; the C‐terminal antibody detected a band at approximately ~75 kDa band (Figure 2a), while the N‐terminal antibody detected a band of ~65 kDa (Figure 2b), this difference in the molecular weight is likely due to the majority of occludin phosphorylation sites residing in the C‐terminal region (Dorfel & Huber, 2012; Raleigh et al., 2011). Additional bands were also detected at ~52, 40 and 23 kDa, these lower molecular weight bands have been described previously as structural monomers or degradation products (Frankowski et al., 2015; McCaffrey et al., 2009). There was little difference in the banding pattern in the different cellular compartments except for the absence of the lower molecular weight bands (37–50 kDa) in the nuclear fraction and the presence of a faint band at ~120 kDa in the nuclear fraction.

### 3.2 In vivo expression in human temporal cortex

**Figure 3:**
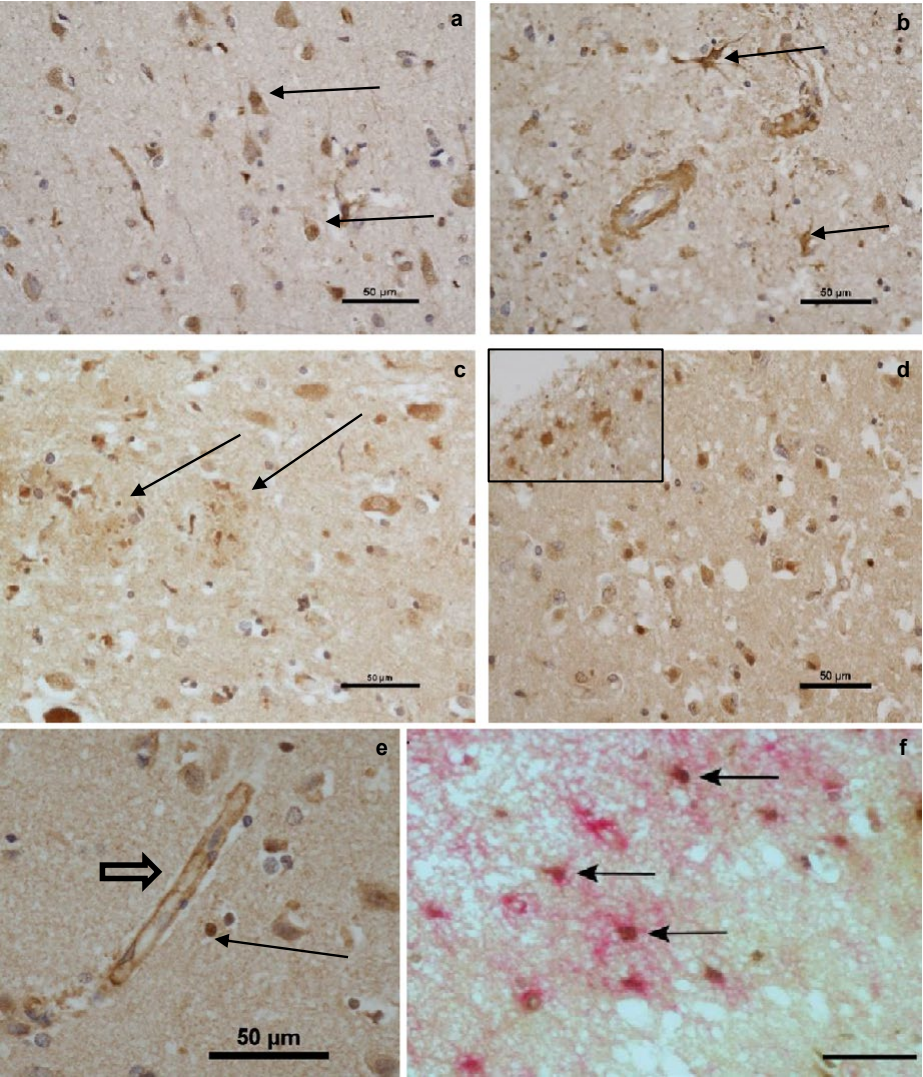
Immunohistochemistry for occludin in human autopsy brain. (a) Occludin pyramidal neuron staining with pyramidal nuclear reactivity indicated (arrows), score 2 (Case 14, MCI). (b) Cytoplasmic astrocyte expression (arrows, Case 21, AD). (c) Occludin reactivity around a plaque (arrows, Case 29, AD). (d) Occludin up‐regulation around ischaemic focus, inset showing up‐regulation in astrocytes (Case 15, MCI). (e) Occludin immunopositive glial nucleus (arrow). Note several adjacent negative nuclei. An occludin‐labelled capillary is also seen (arrowhead) (Case 11, MCI) (f) Dual labelling immunohistochemistry for occludin (brown) and GFAP (pink). Arrows show occludin positive GFAP‐astrocytes [Colour figure can be viewed at http://wileyonlinelibrary.com]

Expression of occludin was examined in human temporal neocortex from cases with AD, MCI and controls (Figure 3). In addition to the expected expression in endothelial cells, occludin was also expressed in the cytoplasm and nuclei of pyramidal neurons (Figure 3a), which can be recognised on the basis of morphology. Neuronal cytoplasmic expression extended into proximal neurites. Cytoplasmic astrocyte expression was also detected (Figure 3b), and there was also expression in glial nuclei (Figure 3e). Immunohistochemistry also decorated neuritic plaques with a morphology suggesting their localisation in dystrophic neurites (Figure 3c). One case showed up‐regulation of expression of occludin around a focus of cortical ischaemia (Figure 3d). Expression of occludin was also observed in capillaries, consistent with the role of this protein in tight junctions (Figure 3e). Dual‐labelling immunohistochemistry showed co‐localisation of occludin‐positive nuclei with GFAP‐positive cells (Figure 3e), demonstrating the astrocytic expression of this tight junction protein in vivo. Occludin‐GFAP double‐positive cells were not seen in all cases and occludin was not present in all GFAP positive cells.

### 3.3 Mass spectrometry

To further explore nuclear and cytoplasmic, non‐tight junction protein functions, binding partners of occludin were sought and identified using MS. Since we were unable to precipitate occludin from 1321N1 cells using commercial anti‐occludin antibodies we developed a pull‐down assay using GB1‐6His recombinant protein and IgG beads. Full length, N‐terminal and C‐terminal recombinant proteins were expressed in bacteria with the C‐ (271‐522) and N‐ (1‐65) terminal peptides used in a pull‐down assay on lysates from the 1321N1 astrocytoma cell line. Western blots showed that the GB1‐OCLN_N recombinant protein was recognised by its specific antibody and produced a protein band just above 15 kDa (Figure S3). There were also two lower molecular weight bands, which might be truncated forms of occludin. Faint bands around 30 kDa were also visible. The GB1‐OCL N_C recombinant protein was also recognised by its specific antibody and produced a protein band of approximately 45 kDa that corresponds with the size of the protein seen in panel A. Lower molecular weight bands were also present, which again might be truncated forms of occludin. Full‐length occludin peptide, however, proved difficult to purify and was therefore not used in further experiments.

Eluted samples from the pull‐downs were analysed using SDS‐PAGE in conjunction with in gel digestion and MS analysis (Figure S4). Due to the high background in the GB1 controls lanes MS was performed on both GB1 and GB1‐occludin domains and only occludin‐specific hits were retained for the analysis. The results from the MS analysis identified nine potential binding partners using the C‐terminal peptide (Table 3) and 41 proteins with the N‐terminal peptide (Table 4). The full mass spectrometry analysis is provided in Tables S3‐S5 for GB1‐OCLN_C, GB1‐OCLN_N and GB1‐control. Except for actin, none of these proteins were identified in the respective GB1 controls suggesting that they were specific interactions with the molecular domains. However, the identification of indirect binding proteins cannot be discounted and further investigation would be needed to determine which are the direct binding proteins. Although actin was identified in the GB1‐control there was a large difference in the number of peptides identified and overall mascot score, and it has previously been identified as an interacting partner, hence it was retained. Potential binding partners identified included ZO‐1, actin E3 ubiquitin ligases Itch and Nedd4‐2.

**Table 3:** Putative C‐terminal domain occludin binding partners

Accession	Name	Score	Mass (Da)	Description
OCLN_HUMAN	Occludin	1,629	59,505	An integral membrane protein of the TJC
RLA0_HUMAN	60S acidic ribosomal protein P0	202	34,423	A structural component of the ribosome
*ACTB_HUMAN*	*Actin, cytoplasmic 1*	*141*	*42,052*	*A component of the cellular cytoskeleton*
DDX3X_HUMAN	ATP‐dependent RNA helicase DDX3X	87	73,597	Involved in many stages of gene expression including transcription
PCBP1_HUMAN	Poly(rC)‐binding protein 1	78	37,987	Single‐stranded nucleic acid binding protein
G3P_HUMAN	Glyceraldehyde‐3‐phosphate dehydrogenase	77	36,201	Participates in nuclear events including transcription, RNA transport, DNA replication and apoptosis
LMNA_HUMAN	Prelamin‐A/C	58	74,380	A component of the nuclear lamina
HORN_HUMAN	Hornerin	56	283,140	A component of the epidermal cornified cell envelope
*ZO1_HUMAN*	*Tight junction protein ZO‐1*	*56*	*195,682*	*A cytoplasmic scaffolding protein of the TJC*

Note

These proteins were identified by mass spectrometry analysis from a gel produced by a pull‐down protein‐binding assay using GB1‐OCLN_C. Known occludin binding partners are shown in italics.

**Table 4:** Putative N‐terminal domain occludin binding partners

Accession	Name	Score	Mass (Da)	Description
MYH10_HUMAN	Myosin‐10	685	229,827	Involved in cytokinesis
H2B1K_HUMAN	Histone H2B type 1‐K	1,347	13,882	Component of the nucleosome
H32_HUMAN	Histone H3.2	468	15,436	Component of the nucleosome
DHX9_HUMAN	ATP‐dependent RNA helicase A	396	142,181	Unwinds duplex DNA and RNA
PPIB_HUMAN	Peptidyl‐prolyl cis‐trans isomerase B	247	23,785	Facilitates protein folding
SFPQ_HUMAN	Splicing factor, proline‐ and glutamine‐rich	190	76,216	Binds DNA and RNA and is involved in several nuclear processes
NONO_HUMAN	Non‐POU domain‐containing octamer‐binding protein	88	54,311	Binds DNA and RNA and is involved in several nuclear processes
*ITCH_HUMAN*	*E3 ubiquitin‐protein ligase Itchy homologue*	*186*	*103,593*	*An E3 ubiquitin‐protein ligase*
TOP2A_HUMAN	DNA topoisomerase 2‐alpha	185	175,017	Enzyme which catalyses the breakage and reannealing of DNA
TOP2B_HUMAN	DNA topoisomerase 2‐beta	145	184,122	Enzyme which catalyses the breakage and reannealing of DNA
SMCA5_HUMAN	SWI/SNF‐related matrix‐associated actin‐dependent regulator of chromatin subfamily A member 5	181	122,513	ATP‐dependent helicase involved in the remodelling of nucleosomes
HNRPU_HUMAN	Heterogeneous nuclear ribonucleoprotein U	152	91,269	Binds to RNA and is specific to the nucleus
ROA3_HUMAN	Heterogeneous nuclear ribonucleoprotein A3	140	39,799	RNA‐binding protein possibly involved in mRNA nuclear export
ML12A_HUMAN	Myosin regulatory light chain 12A	134	19,839	Involved in cytokinesis
ROA2_HUMAN	Heterogeneous nuclear ribonucleoproteins A2/B1	133	37,464	RNA‐binding protein possibly involved in mRNA nuclear export
U520_HUMAN	U5 small nuclear ribonucleoprotein 200 kDa helicase	131	246,006	RNA helicase
NOP56_HUMAN	Nucleolar protein 56	119	66,408	Involved in ribosomal subunit biogenesis
HNRPL_HUMAN	Heterogeneous nuclear ribonucleoprotein L	114	64,720	Splicing factor
H14_HUMAN	Histone H1.4	112	21,852	Histone
NPM_HUMAN	Nucleophosmin	112	32,726	Multi‐functional protein involved in ribosome biogenesis and transport
RS14_HUMAN	40S ribosomal protein S14	96	16,434	Component of the ribosome
NOP58_HUMAN	Nucleolar protein 58	95	60,054	Involved in ribosomal subunit biogenesis
PSIP1_HUMAN	PC4 and SFRS1‐interacting protein	95	60,181	Transcriptional coactivator
DCD_HUMAN	Dermcidin	92	11,391	An anti‐microbial peptide
*NEDD4_HUMAN*	*E3 ubiquitin‐protein ligase NEDD4*	*83*	*150,276*	*An E3 ubiquitin‐protein ligase*
RBMX_HUMAN	RNA‐binding motif protein, X chromosome	81	42,306	RNA‐binding protein
DDX21_HUMAN	Nucleolar RNA helicase 2	79	87,804	RNA helicase
ARPC4_HUMAN	Actin‐related protein 2/3 complex subunit 4	72	19,768	An actin‐binding component which is involved in actin polymerisation
RL40_HUMAN	Ubiquitin‐60S ribosomal protein L40	72	15,004	Component of the ribosome
U5S1_HUMAN	116 kDa U5 small nuclear ribonucleoprotein component	70	110,336	A component required for pre‐RNA splicing
RS6_HUMAN	40S ribosomal protein S6	68	28,834	Ribosomal Protein
DHX15_HUMAN	Putative pre‐mRNA‐splicing factor ATP‐dependent RNA helicase DHX15	66	91,673	A factor involved in pre‐mRNA processing
H2AY_HUMAN	Core histone macro‐H2A.1	66	39,764	Histone
RS25_HUMAN	40S ribosomal protein S25	65	13,791	Ribosomal Protein
ILF2_HUMAN	Interleukin enhancer‐binding factor 2	63	43,263	Appears to interact with ILF3 and may regulate IL2 gene transcription
HNRPD_HUMAN	Heterogeneous nuclear ribonucleoprotein D0	59	38,581	RNA‐binding protein
NCBP1_HUMAN	Nuclear cap‐binding protein subunit 1	56	92,864	A component in complexes involved in RNA processing
ELAV1_HUMAN	ELAV‐like protein 1	55	36,240	RNA‐binding protein
RS18_HUMAN	40S ribosomal protein S18	50	17,708	Ribosomal Protein

Note

Proteins identified by mass spectrometry analysis from a gel produced by a pull‐down protein‐binding assay conducted using GB1‐OCLN_N. Known occludin binding partners are shown in italics.

## 4 DISCUSSION

We examined the expression of occludin in human astrocytes in vitro and in human tissue in vivo. The subcellular localisation pattern, combined with the protein‐binding partners identified by proteomic analysis, suggest non‐tight junction‐related functions in these neuroepithelial cells. Nuclear and cytoplasmic expression was demonstrated in the 1321N1 astrocytoma cell line. Cell lines such as this are convenient to use because of their growth characteristics, and we therefore used this line to obtain adequate amounts of protein lysates for the binding partner assays. However, this line is neoplasm‐derived and, so as to ensure that this is not a cell‐line effect, we also confirmed expression in well characterised human primary astrocyte cells (Garwood et al., 2015) and in human post‐mortem brain tissue.

Previous studies demonstrated expression of occludin in rodent derived astrocytes and neurons in culture and human foetal cortical astrocytes (Duffy, John, Lee, Brosnan, & Spray, 2000), with expression varying with differentiation state (Bauer et al., 1999). Expression has also been described in neurons, astrocytes and oligodendrocytes in human brain (Romanitan et al., 2007).

Here, nuclear expression of occludin was a consistent feature both in vitro and in vivo. Although non‐tight junction functions have previously been described for the ZO‐proteins, suggesting that, as a group, these proteins have other important cellular roles, there is less evidence relating to potential non‐tight junction roles of occludin. Aside from its role as a tight junction protein occludin regulates centrosome separation and mitotic entry in Madin‐Darby canine kidney cells (MDCK) (Runkle, Sundstrom, Runkle, Liu, & Antonetti, 2011), and recently a novel role for occludin in the regulation of retinal endothelial proliferation and angiogenesis has been described (Liu et al., 2016). As well as bands in the expected molecular weight range for occludin additional smaller molecular weight bands were detected. These lower molecular weight occludin “isoforms” have been described previously in endothelial cells (Frankowski et al., 2015; McCaffrey et al., 2009) and are detected by multiple occludin antibodies (McCaffrey et al., 2007). It is not known if these smaller isoforms of occludin have any functional significance.

Similar patterns of expression were observed in neuroepithelial cells in human post‐mortem tissue, demonstrating that these subcellular localisations are not artefacts of cell culture. As our aim here was to demonstrate that cell culture patterns were good models of the in vivo state, we did not quantify variation in expression in the human tissue, but there is evidence that non‐tight junction expression may vary with disease state. Several studies have found that occludin and certain claudins are increased in Alzheimer's disease and vascular dementia (Romanitan et al., 2007, 2010; Spulber, Bogdanovic, Romanitan, Bajenaru, & Popescu, 2012). In contrast, using microarrays coupled with laser‐capture microdissection, we previously found a decline in RNA expression for occludin specifically in astrocytes with Alzheimer's neuropathology progression (Simpson et al., 2011). However, protein expression may not parallel that of mRNA. It is possible that other pathological factors, such as oxidative damage may contribute to the variation in expression of these proteins, and pathological factors driving alteration of these proteins remain to be fully defined. An interesting observation in one case in the current study was up‐regulation of occludin around an incidental area of infarction. This observation may suggest that expression is altered in response to ischaemia/infarction and is worth further investigation. Expression in the neuritic processes around plaques was a notable feature.

To further define possible functions of occludin in astrocytes, we undertook a proteomic analysis using MS. We were unable to precipitate occludin directly from 1321N1 or ScienCell astrocytes. We therefore used recombinant N‐ and C‐terminal occludin peptides to pull‐down putative binding partners. MS has been previously used to identify proteins in tight junction complexes in MDCK‐II cells (Fredriksson et al., 2015). We have now used this method to identify binding proteins in non‐tight junction bearing cells. Nine binding partners were identified with the C‐terminal peptide, and 41 with the N‐terminal. These included proteins that have previously been shown to bind to occludin. Both ZO‐1 and actin, which were identified in the GB1‐OCLN_C pull‐down protein‐binding assay, are known to interact with occludin at the C‐terminal domain (Li, Fanning, Anderson, & Lavie, 2005; Wittchen, Haskins, & Stevenson, 1999). Similarly the E3 ubiquitin ligases Itch and Nedd4‐2, which have previously been shown to interact with occludin at the N‐terminal domain (Raikwar, Vandewalle, & Thomas, 2010; Traweger et al., 2002), were identified as occludin binding partners in the GB1‐OCLN_N pull‐down protein‐binding assay. Thus, our method was able to identify previously known binding partners.

Existing literature suggests binding of proteins that are associated with nuclear and cytoplasmic functions. ELL homology in the C‐terminus suggests transcriptional processes, and interactions with epsin‐1, and dynamin have been described (Li et al., 2005; Liu et al., 2010; Murakami, Felinski, & Antonetti, 2009). The binding partners identified in the present proteomic study suggest novel functions particularly in RNA metabolism and in nuclear functions.

DEAD‐box helicase 3, X‐linked (DDX3X) was identified as a binding partner. DDX3X is a 662 amino acid, 73 kDa nucleocytoplasmic ATP‐dependent RNA helicase (Jankowsky, 2011). DEAD‐box helicases bind to RNA and ATP through motifs located in the helicase core domain and are involved in RNA metabolism (Putnam & Jankowsky, 2013). DDX3X interacts with a nuclear export protein and component of the mRNP complex known as tip‐associated protein (TAP) (Guzik et al., 2001; Lai, Lee, & Tarn, 2008). DDX3X also directly interacts with mRNA and there is also evidence to suggest that DDX3X is involved in translation initiation and the functional assembly of 80S ribosomes (Geissler, Golbik, & Behrens, 2012; Lai et al., 2008). Interaction with DDX3X and other binding proteins therefore suggests that occludin may have roles in RNA metabolism.

Several heterogeneous nuclear ribonucleoproteins (hnRNPs) were also identified as putative occludin N‐terminal domain binding partners. These proteins interact with pre‐mRNAs and function as components of RNP complexes facilitating RNA metabolic processes including mRNA export (Geuens, Bouhy, & Timmerman, 2016). hnRNP A2/B1 and hnRNPA3 were identified as binding partners. These proteins contain a nuclear transport signal (M9 motif) (Bogerd et al., 1999; Ma et al., 2002; Michael, Choi, & Dreyfuss, 1995), suggesting a role in the translocation of occludin between the nucleus and cytoplasm in astrocytes. Further analysis was performed using the Search Tool for the Retrieval of Interating Genes/Proteins (STRING) to identify if any of the proteins identified through the MS were predicted to interact. Only ELAVL1, an RNA‐binding protein that binds to the 3′‐UTR region of mRNAs to increase their stability, was present in both the MS and the STRING analysis. However this lack of overlap is perhaps not unsurprising as STRING predicts associations based on current literature as well as computational prediction methods, and almost all of the research to date on occludin has been focused on its role in tight junctions.

In conclusion, our data strongly suggest occludin has important non‐tight junction‐related roles in neuroepithelial cells in vivo. Candidate protein‐binding partners identified in this study suggest that these functions may relate to RNA metabolism and nuclear functions, processes that are altered in a variety of human neurodegenerative disorders. Defining the role of alterations in the expression of occludin and its interactions with proteins in these key metabolic pathways is therefore an important question for future studies of neuroglial cell function and neurodegeneration.

## CONFLICTS OF INTEREST

The authors declare no competing financial interests.

## DATA ACCESSIBILITY

List of genes and anonymised case information are provided in the supplementary material. Other information can be available on request.

## AUTHOR CONTRIBUTIONS

SVM planned and carried out experiments and performed most of the literature search. The study was conceived by SBW. The first draft of the paper was written by SBW, CJG and JES. All of the authors contributed to supervision of the project. CJG, LJ and IV‐V contributed to cell culture experiments and LC performed cell fractionations. SBW, PGI and JES oversaw histological studies. PRH oversaw molecular methods. GMH supervised and planned the protein extraction experiments and SRM contributed to these experiments. MJD supervised, and SVM and TCM performed, the mass spectrometry analysis. All of the authors contributed to the final version of the manuscript.
