# Supplementary material for: Proteomic and cellular localisation studies suggest non‐tight junction cytoplasmic and nuclear roles for occludin in astrocytes
Source: Eur J Neurosci. 2018 May 30;47(12):1444–56. doi: 10.1111/ejn.13933 (PMC6079634; doi:10.1111/ejn.13933)
Supplement: Supplementary file 2 [file EJN-47-1444-s002.pdf]

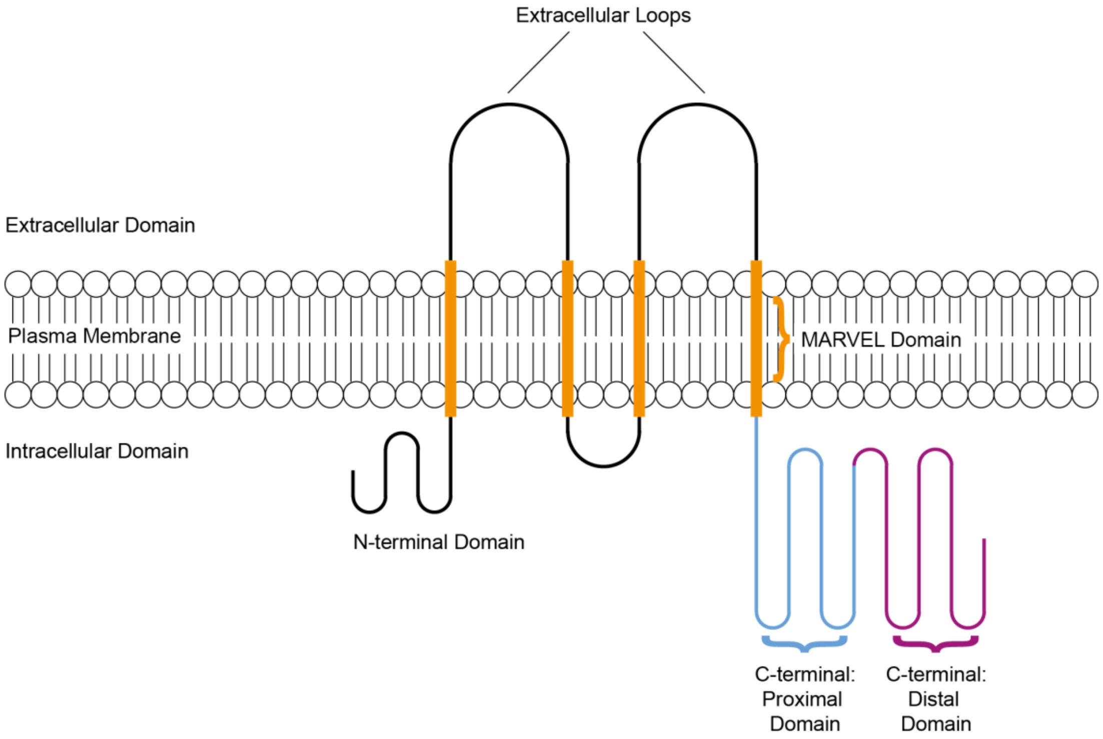

Supplementary Figure 1 Schematic depicting occludin protein structure consisting of the transmembrane MARVEL domain (orange), two extracellular loops, a short intracellular loop, a short cytoplasmic N-terminal domain and a long cytoplasmic C-terminal domain. The C-terminal domain itself consists of the membrane-proximal domain (blue) and the membrane-distal domain (purple**).**

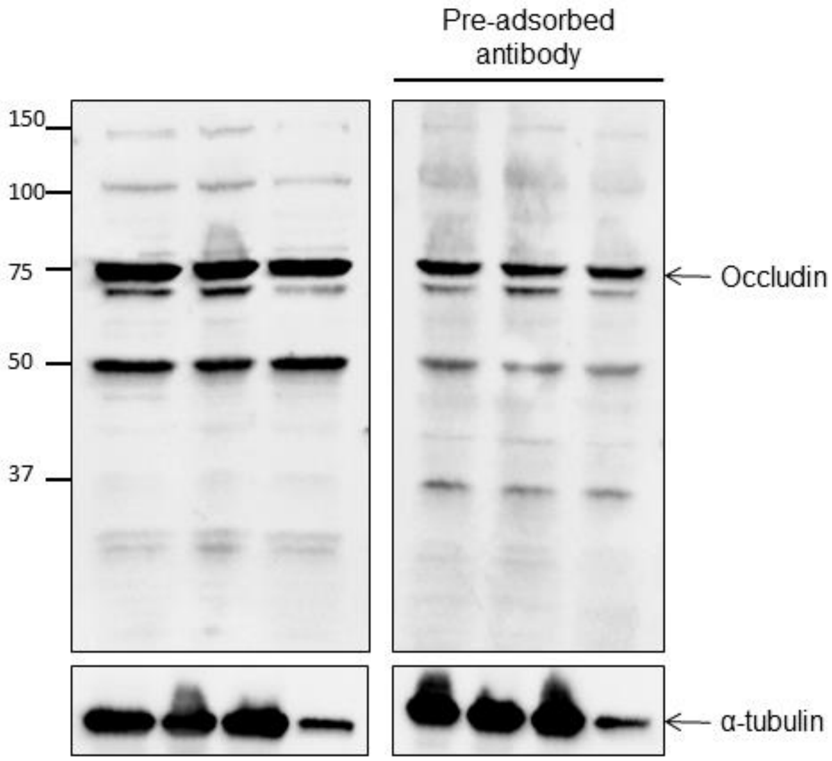

Supplementary Figure 2: Specificity of the Occludin antibody (Thermofisher antibody 71-1500) was confirmed by pre-adsorbing the primary antibody with recombinant C-terminal occludin. 1.25μg occlusion antbody was incubated with 125 μg recombinant C-terminal occludin O/N at 4°C. 1321N1 cell lysates (~40μg loaded) were electrophoresed on a 10% resolving gel and proteins transferred onto a nitrocellulose membrane. After transfer the membrane was divided into 2, one half was incubated O/N in occludin antibody, the other was incubated O/N in the pre-adsorbed occludin antibody. After incubation in primary antibody membranes were washed and incubated in the same HRP-conjugated secondary antibody and then developed using the same ECL solution (simultaneously). Molecular weight markers are shown in kDa.

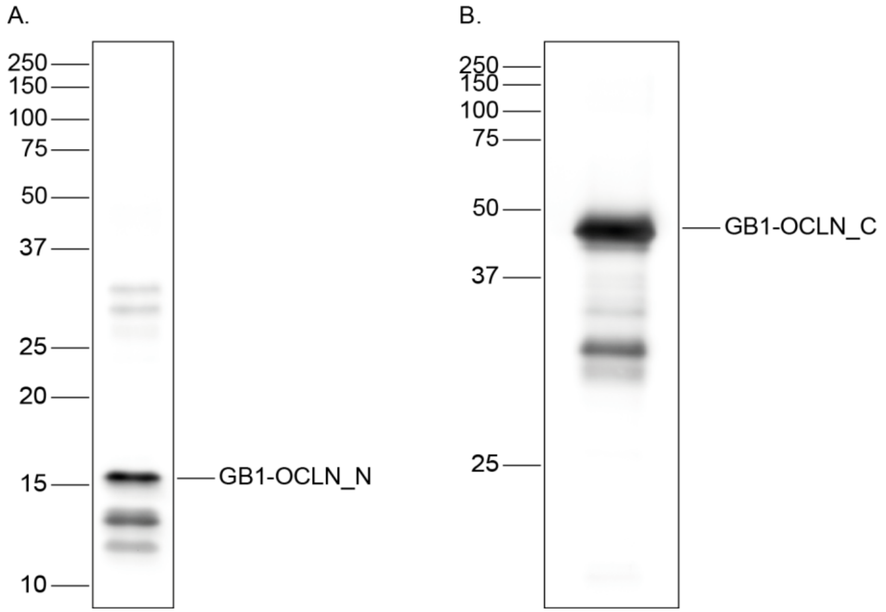

Supplementary figure 3: Western blots of GB1-OCLN_N and GB1-OCLN_C Western blot of GB1-OCLN_N probed with anti-occludin rabbit monoclonal antibody (ab167161) and (B) western blot of GB1-OCLN_C probed with anti-occludin rabbit polyclonal antibody (71-1500). Molecular weight markers are indicated (kDa).

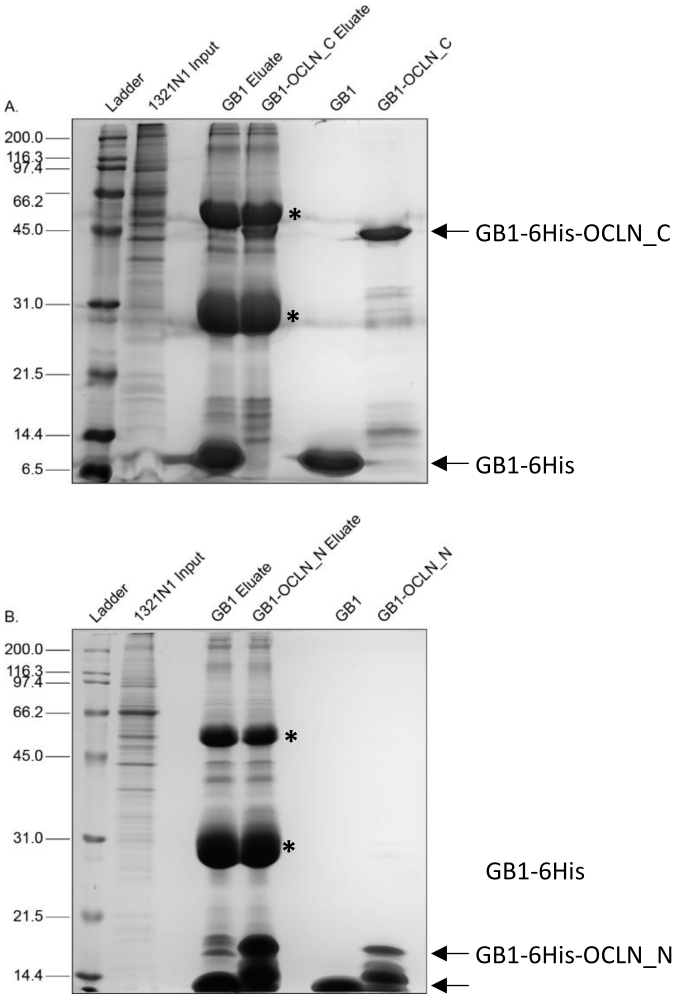

Supplementary figure 4: GB1-OCLN_C and GB1-OCLN_N pull-down protein binding assay gels sent for Mass Spectrometry Analysis A) GB1-OCLN_C pull-down protein binding assay gel. B) GB1-OCLN_N pull-down protein binding assay gel. Both gels were stained with Instant*Blue* coomassie. These experiments were conducted using a 12% resolving gel with a 4% stacking gel, * represent heavy and light IgG bands. Molecular weight markers are indicated (kDa).

Supplementary Table 1: Cases Used for Histological Analysis

Case	Group	Age (yrs)	Sex	PMI (hrs)	Fixation (wks)	Braak NFT Stage
1	C	58	F	39	5	0
2	C	72	F	27	9	1
3	C	59	F	19	11	0
4	C	74	F	67	14	1
5	C	55	M	41	14	0
6	C	70	M	72	11	0
7	C	78	F	34	8	0
8	C	83	F	24	8	1
9	C	73	M	25	9	0
10	C	74	F	49	12	1
11	MCI	105	F	42	NA	4
12	MCI	91	F	17	NA	4
13	MCI	75	M	82	25	4
14	MCI	86	F	75	7	4
15	MCI	88	F	22	10	3
16	MCI	81	M	82	9	3
17	MCI	98	F	59	9	3
18	MCI	89	F	34	8	3
19	MCI	95	F	66	8	3
20	AD	84	F	47	NA	6
21	AD	77	F	63	6	6
22	AD	93	F	53	10	6
23	AD	99	F	71	17	6
24	AD	83	M	12	NA	6
25	AD	80	F	32	17	6
26	AD	86	F	69	`5	6
27	AD	88	M	84	10	6
28	AD	78	M	37	14	6
29	AD	78	F	40	12	6

Abbreviations: C Controls, MCI mild cognitive impairment, AD Alzheimer's disease. F female, M male. PMI post mortem interval. NA not available. NFT neurofibrillary tangle.

Supplementary Table 2: Group Characteristics

Group	Mean Age (yrs)*	Mean PMI (hrs)	Mean Fixation (wks)
**Control**	69.6	39.7	10.1
**MCI**	87.4	60.0	10.9
**AD**	84.9	56.13	12.6

*The differences in ages between groups is significant (Kruskal-Wallis test, p<0.001). No significant difference for PMI or fixation.

Supplementary Table 3: OCLN_C Raw Mass Spectrometry Data:

Filters: Number of significant unique sequences > 1

Accession	Score	Mass	Num. of matches	Num. of significant matches	Num. of sequences	Num. of significant sequences	emPAI	
IGHG1_HUMAN	4129	36596	360	185	21	15	6.56	Ig gamma-1 chain C region OS=Homo sapiens GN=IGHG1 PE=1 SV=1
IGHG3_HUMAN	2411	42287	307	138	21	16	4.33	Ig gamma-3 chain C region OS=Homo sapiens GN=IGHG3 PE=1 SV=2
IGHG2_HUMAN	2029	36505	270	116	16	12	3.69	Ig gamma-2 chain C region OS=Homo sapiens GN=IGHG2 PE=1 SV=2
K2C1_HUMAN	2527	66170	253	114	42	23	2.64	Keratin, type II cytoskeletal 1 OS=Homo sapiens GN=KRT1 PE=1 SV=6
K22E_HUMAN	1198	65678	102	47	34	18	1.8	Keratin, type II cytoskeletal 2 epidermal OS=Homo sapiens GN=KRT2 PE=1 SV=2
K2C5_HUMAN	236	62568	49	11	28	7	0.58	Keratin, type II cytoskeletal 5 OS=Homo sapiens GN=KRT5 PE=1 SV=3
IGKC_HUMAN	2105	11773	174	110	8	6	16.67	Ig kappa chain C region OS=Homo sapiens GN=IGKC PE=1 SV=1
OCLN_HUMAN	1629	59505	161	83	19	13	2.51	Occludin OS=Homo sapiens GN=OCLN PE=1 SV=1
K1C9_HUMAN	1482	62255	109	58	27	15	1.64	Keratin, type I cytoskeletal 9 OS=Homo sapiens GN=KRT9 PE=1 SV=3
LAC2_HUMAN	1431	11458	140	73	8	6	13.23	Ig lambda-2 chain C regions OS=Homo sapiens GN=IGLC2 PE=1 SV=1
IGLL5_HUMAN	1211	23391	126	54	10	4	1.44	Immunoglobulin lambda-like polypeptide 5 OS=Homo sapiens GN=IGLL5 PE=2 SV=2
K1C10_HUMAN	809	59020	80	33	26	17	1.79	Keratin, type I cytoskeletal 10 OS=Homo sapiens GN=KRT10 PE=1 SV=6

K1C14_HUMAN	233	51872	42	9	25	9	0.85	Keratin, type I cytoskeletal 14 OS=Homo sapiens GN=KRT14 PE=1 SV=4
HV307_HUMAN	337	13773	44	15	10	4	1.69	Ig heavy chain V-III region CAM OS=Homo sapiens PE=1 SV=1
H4_HUMAN	255	11360	20	14	9	6	4.97	Histone H4 OS=Homo sapiens GN=HIST1H4A PE=1 SV=2
ALBU_HUMAN	205	71317	49	18	16	5	0.35	Serum albumin OS=Homo sapiens GN=ALB PE=1 SV=2
KV305_HUMAN	203	11853	25	10	5	2	0.77	Ig kappa chain V-III region WOL OS=Homo sapiens PE=1 SV=1
RLA0_HUMAN	202	34423	19	10	10	6	1.05	60S acidic ribosomal protein P0 OS=Homo sapiens GN=RPLP0 PE=1 SV=1
KV203_HUMAN	178	12162	13	7	4	2	0.74	Ig kappa chain V-II region MIL OS=Homo sapiens PE=1 SV=1
HNRPM_HUMAN	146	77749	67	7	40	7	0.38	Heterogeneous nuclear ribonucleoprotein M OS=Homo sapiens GN=HNRNPM PE=1 SV=3
ACTB_HUMAN	141	42052	13	5	11	4	0.4	Actin, cytoplasmic 1 OS=Homo sapiens GN=ACTB PE=1 SV=1
HSP7C_HUMAN	128	71082	38	8	27	8	0.49	Heat shock cognate 71 kDa protein OS=Homo sapiens GN=HSPA8 PE=1 SV=1
GRP78_HUMAN	54	72402	36	4	25	4	0.22	78 kDa glucose-regulated protein OS=Homo sapiens GN=HSPA5 PE=1 SV=2
HV310_HUMAN	126	13671	41	15	6	3	1.11	Ig heavy chain V-III region HIL OS=Homo sapiens PE=1 SV=1
KV402_HUMAN	88	12746	17	3	6	2	0.7	Ig kappa chain V-IV region Len OS=Homo sapiens PE=1 SV=2
DDX3X_HUMAN	87	73597	28	4	23	4	0.21	ATP-dependent RNA helicase DDX3X OS=Homo sapiens GN=DDX3X PE=1 SV=3
RL11_HUMAN	85	20468	12	3	10	3	0.67	60S ribosomal protein L11 OS=Homo sapiens GN=RPL11 PE=1 SV=2
PCBP1_HUMAN	78	37987	20	2	14	2	0.2	Poly(rC)-binding protein 1 OS=Homo sapiens GN=PCBP1 PE=1 SV=2
G3P_HUMAN	77	36201	20	2	15	2	0.22	Glyceraldehyde-3-phosphate dehydrogenase OS=Homo sapiens GN=GAPDH PE=1 SV=3
H31T_HUMAN	70	15613	16	2	9	2	0.55	Histone H3.1t OS=Homo sapiens GN=HIST3H3 PE=1 SV=3
LMNA_HUMAN	58	74380	46	5	31	4	0.27	Prelamin-A/C OS=Homo sapiens GN=LMNA PE=1 SV=1
HORN_HUMAN	56	283140	52	3	35	3	0.04	Hornerin OS=Homo sapiens GN=HRNR PE=1 SV=2
ZO1_HUMAN	56	195682	47	3	34	3	0.06	Tight junction protein ZO-1 OS=Homo sapiens GN=TJP1 PE=1 SV=3
H2B1B_HUMAN	45	13942	13	2	6	2	0.63	Histone H2B type 1-B OS=Homo sapiens GN=HIST1H2BB PE=1 SV=2
GRP75_HUMAN	45	73920	38	3	32	3	0.16	Stress-70 protein, mitochondrial OS=Homo sapiens GN=HSPA9 PE=1 SV=2
RS6_HUMAN	38	28834	11	2	7	2	0.28	40S ribosomal protein S6 OS=Homo sapiens GN=RPS6 PE=1 SV=1
RL23_HUMAN	30	14970	10	2	7	2	0.58	60S ribosomal protein L23 OS=Homo sapiens GN=RPL23 PE=1 SV=1
RL27_HUMAN	28	15788	4	2	4	2	0.55	60S ribosomal protein L27 OS=Homo sapiens GN=RPL27 PE=1 SV=2
TITIN_HUMAN	16	3842904	707	2	536	2	0	Titin OS=Homo sapiens GN=TTN PE=1 SV=4

Supplementary Table 4: OCLN_N Raw Mass Spectrometry Data

Filters: Number of significant unique sequences > 1

Accession	Score	Mass	Num. of matches	Num. of significant matches	Num. of sequences	Num. of significant sequences	emPAI	
MYH10_HUMAN	685	229827	76	48	46	30	0.87	Myosin-10 OS=Homo sapiens GN=MYH10 PE=1 SV=3
H2B1K_HUMAN	1347	13882	111	85	12	10	215.4	Histone H2B type 1-K OS=Homo sapiens GN=HIST1H2BK PE=1 SV=3
H4_HUMAN	613	11360	62	44	7	7	35.27	Histone H4 OS=Homo sapiens GN=HIST1H4A PE=1 SV=2
H32_HUMAN	468	15436	69	32	10	6	7.89	Histone H3.2 OS=Homo sapiens GN=HIST2H3A PE=1 SV=3
DHX9_HUMAN	396	142181	37	23	14	11	0.43	ATP-dependent RNA helicase A OS=Homo sapiens GN=DHX9 PE=1 SV=4
HNRPC_HUMAN	295	33707	22	19	8	5	1.21	Heterogeneous nuclear ribonucleoproteins C1/C2 OS=Homo sapiens GN=HNRNPC PE=1 SV=4
PPIB_HUMAN	247	23785	24	11	9	4	2.06	Peptidyl-prolyl cis-trans isomerase B OS=Homo sapiens GN=PPIB PE=1 SV=2
SFPQ_HUMAN	190	76216	22	14	8	6	0.58	Splicing factor, proline- and glutamine-rich OS=Homo sapiens GN=SFPQ PE=1 SV=2
NONO_HUMAN	88	54311	10	5	7	4	0.33	Non-POU domain-containing octamer-binding protein OS=Homo sapiens GN=NONO PE=1 SV=4
ITCH_HUMAN	186	103593	19	13	12	8	0.4	E3 ubiquitin-protein ligase Itchy homolog OS=Homo sapiens GN=ITCH PE=1 SV=2
TOP2A_HUMAN	185	175017	24	14	18	13	0.33	DNA topoisomerase 2-alpha OS=Homo sapiens GN=TOP2A PE=1 SV=3

TOP2B_HUMAN	145	184122	13	11	12	10	0.23	DNA topoisomerase 2-beta OS=Homo sapiens GN=TOP2B PE=1 SV=3
SMCA5_HUMAN	181	122513	15	5	9	3	0.1	SWI/SNF-related matrix-associated actin-dependent regulator of chromatin subfamily A member 5 OS=Homo sapiens GN=SMARCA5 PE=1 SV=1
HNRPU_HUMAN	152	91269	15	9	10	6	0.29	Heterogeneous nuclear ribonucleoprotein U OS=Homo sapiens GN=HNRNPU PE=1 SV=6
ROA3_HUMAN	140	39799	10	9	5	5	0.62	Heterogeneous nuclear ribonucleoprotein A3 OS=Homo sapiens GN=HNRNPA3 PE=1 SV=2
ML12A_HUMAN	134	19839	7	6	4	4	1.59	Myosin regulatory light chain 12A OS=Homo sapiens GN=MYL12A PE=1 SV=2
ROA2_HUMAN	133	37464	10	5	5	2	0.23	Heterogeneous nuclear ribonucleoproteins A2/B1 OS=Homo sapiens GN=HNRNPA2B1 PE=1 SV=2
U520_HUMAN	131	246006	12	6	8	3	0.07	U5 small nuclear ribonucleoprotein 200 kDa helicase OS=Homo sapiens GN=SNRNP200 PE=1 SV=2
NOP56_HUMAN	119	66408	14	7	11	7	0.5	Nucleolar protein 56 OS=Homo sapiens GN=NOP56 PE=1 SV=4
HNRPL_HUMAN	114	64720	18	9	9	5	0.35	Heterogeneous nuclear ribonucleoprotein L OS=Homo sapiens GN=HNRNPL PE=1 SV=2
H14_HUMAN	112	21852	9	5	8	4	1	Histone H1.4 OS=Homo sapiens GN=HIST1H1E PE=1 SV=2
NPM_HUMAN	112	32726	8	5	3	2	0.42	Nucleophosmin OS=Homo sapiens GN=NPM1 PE=1 SV=2
RS14_HUMAN	96	16434	6	6	3	3	1.49	40S ribosomal protein S14 OS=Homo sapiens GN=RPS14 PE=1 SV=3
NOP58_HUMAN	95	60054	15	6	11	5	0.47	Nucleolar protein 58 OS=Homo sapiens GN=NOP58 PE=1 SV=1
PSIP1_HUMAN	95	60181	28	7	9	4	0.38	PC4 and SFRS1-interacting protein OS=Homo sapiens GN=PSIP1 PE=1 SV=1
DCD_HUMAN	92	11391	8	5	4	4	2.65	Dermcidin OS=Homo sapiens GN=DCD PE=1 SV=2
NEDD4_HUMAN	83	150276	20	8	9	4	0.14	E3 ubiquitin-protein ligase NEDD4 OS=Homo sapiens GN=NEDD4 PE=1 SV=4
RBMX_HUMAN	81	42306	8	3	7	3	0.31	RNA-binding motif protein, X chromosome OS=Homo sapiens GN=RBMX PE=1 SV=3
DDX21_HUMAN	79	87804	10	5	8	4	0.19	Nucleolar RNA helicase 2 OS=Homo sapiens GN=DDX21 PE=1 SV=5
ARPC4_HUMAN	72	19768	6	5	5	4	1.15	Actin-related protein 2/3 complex subunit 4 OS=Homo sapiens GN=ARPC4 PE=1 SV=3
RL40_HUMAN	72	15004	6	3	3	2	0.65	Ubiquitin-60S ribosomal protein L40 OS=Homo sapiens GN=UBA52 PE=1 SV=2
U5S1_HUMAN	70	110336	5	3	5	3	0.11	116 kDa U5 small nuclear ribonucleoprotein component OS=Homo sapiens GN=EFTUD2 PE=1 SV=1
RS6_HUMAN	68	28834	3	2	3	2	0.3	40S ribosomal protein S6 OS=Homo sapiens GN=RPS6 PE=1 SV=1
DHX15_HUMAN	66	91673	9	7	7	5	0.29	Putative pre-mRNA-splicing factor ATP-dependent RNA helicase DHX15 OS=Homo sapiens GN=DHX15 PE=1 SV=2
H2AY_HUMAN	66	39764	8	5	6	4	0.47	Core histone macro-H2A.1 OS=Homo sapiens GN=H2AFY PE=1 SV=4
RS25_HUMAN	65	13791	6	4	2	2	1.25	40S ribosomal protein S25 OS=Homo sapiens GN=RPS25 PE=1 SV=1

ILF2_HUMAN	63	43263	5	4	2	2	0.19	Interleukin enhancer-binding factor 2 OS=Homo sapiens GN=ILF2 PE=1 SV=2
HNRPD_HUMAN	59	38581	4	4	2	2	0.22	Heterogeneous nuclear ribonucleoprotein D0 OS=Homo sapiens GN=HNRNPD PE=1 SV=1
NCBP1_HUMAN	56	92864	3	2	3	2	0.09	Nuclear cap-binding protein subunit 1 OS=Homo sapiens GN=NCBP1 PE=1 SV=1
ELAV1_HUMAN	55	36240	4	2	4	2	0.24	ELAV-like protein 1 OS=Homo sapiens GN=ELAVL1 PE=1 SV=2
RS18_HUMAN	50	17708	5	2	4	2	0.53	40S ribosomal protein S18 OS=Homo sapiens GN=RPS18 PE=1 SV=3

Supplementary Table 5: GB-1 control Raw Mass Spectrometry Data

Filters:: Number of significant unique sequences > 1

Accession	Score	Mass	Num. of matches	Num. of significant matches	Num. of sequences	Num. of significant sequences	emPAI	
IGHG1_HUMAN	3695	36596	306	156	20	11	4.1	Ig gamma-1 chain C region OS=Homo sapiens GN=IGHG1 PE=1 SV=1
IGHG3_HUMAN	1780	42287	269	114	19	13	3.47	Ig gamma-3 chain C region OS=Homo sapiens GN=IGHG3 PE=1 SV=2
IGHG2_HUMAN	1712	36505	268	123	17	9	2.84	Ig gamma-2 chain C region OS=Homo sapiens GN=IGHG2 PE=1 SV=2
IGKC_HUMAN	2705	11773	199	134	6	6	16.41	Ig kappa chain C region OS=Homo sapiens GN=IGKC PE=1 SV=1
K2C1_HUMAN	2151	66170	240	106	37	17	1.62	Keratin, type II cytoskeletal 1 OS=Homo sapiens GN=KRT1 PE=1 SV=6
K22E_HUMAN	737	65678	120	37	36	8	0.54	Keratin, type II cytoskeletal 2 epidermal OS=Homo sapiens GN=KRT2 PE=1 SV=2
K2C6A_HUMAN	157	60293	68	7	28	4	0.26	Keratin, type II cytoskeletal 6A OS=Homo sapiens GN=KRT6A PE=1 SV=3
K2C5_HUMAN	133	62568	45	7	23	4	0.25	Keratin, type II cytoskeletal 5 OS=Homo sapiens GN=KRT5 PE=1 SV=3
LAC2_HUMAN	1493	11458	168	77	8	6	9.46	Ig lambda-2 chain C regions OS=Homo sapiens GN=IGLC2 PE=1 SV=1
IGLL5_HUMAN	925	23391	145	48	9	4	1.1	Immunoglobulin lambda-like polypeptide 5 OS=Homo sapiens GN=IGLL5 PE=2 SV=2
K1C9_HUMAN	1148	62255	102	58	19	7	0.58	Keratin, type I cytoskeletal 9 OS=Homo sapiens GN=KRT9 PE=1 SV=3

K1C10_HUMAN	766	59020	109	42	26	9	0.82	Keratin, type I cytoskeletal 10 OS=Homo sapiens GN=KRT10 PE=1 SV=6
K1C16_HUMAN	254	51578	47	12	23	10	0.98	Keratin, type I cytoskeletal 16 OS=Homo sapiens GN=KRT16 PE=1 SV=4
K1C14_HUMAN	228	51872	48	11	22	8	0.72	Keratin, type I cytoskeletal 14 OS=Homo sapiens GN=KRT14 PE=1 SV=4
K1H1_HUMAN	85	48633	29	5	9	3	0.24	Keratin, type I cuticular Ha1 OS=Homo sapiens GN=KRT31 PE=2 SV=3
HV305_HUMAN	429	13332	59	22	7	3	1.76	Ig heavy chain V-III region BRO OS=Homo sapiens PE=1 SV=1
ACTG_HUMAN	400	42108	44	17	21	9	1.12	Actin, cytoplasmic 2 OS=Homo sapiens GN=ACTG1 PE=1 SV=1
ALBU_HUMAN	397	71317	67	22	16	6	0.35	Serum albumin OS=Homo sapiens GN=ALB PE=1 SV=2
HSP7C_HUMAN	324	71082	52	14	32	11	0.73	Heat shock cognate 71 kDa protein OS=Homo sapiens GN=HSPA8 PE=1 SV=1
GRP78_HUMAN	139	72402	36	8	24	7	0.41	78 kDa glucose-regulated protein OS=Homo sapiens GN=HSPA5 PE=1 SV=2
KV203_HUMAN	206	12162	12	9	3	2	0.74	Ig kappa chain V-II region MIL OS=Homo sapiens PE=1 SV=1
KV305_HUMAN	167	11853	30	9	3	2	0.76	Ig kappa chain V-III region WOL OS=Homo sapiens PE=1 SV=1
P5CS_HUMAN	166	87989	37	9	24	9	0.44	Delta-1-pyrroline-5-carboxylate synthase OS=Homo sapiens GN=ALDH18A1 PE=1 SV=2
H2BFS_HUMAN	160	13936	24	5	11	2	0.62	Histone H2B type F-S OS=Homo sapiens GN=H2BFS PE=1 SV=2
H4_HUMAN	152	11360	20	7	13	5	3.4	Histone H4 OS=Homo sapiens GN=HIST1H4A PE=1 SV=2
H2A1B_HUMAN	119	14127	18	8	7	2	1.62	Histone H2A type 1-B/E OS=Homo sapiens GN=HIST1H2AB PE=1 SV=2
GRP75_HUMAN	115	73920	38	5	32	5	0.27	Stress-70 protein, mitochondrial OS=Homo sapiens GN=HSPA9 PE=1 SV=2
KRT82_HUMAN	91	57985	23	3	17	2	0.13	Keratin, type II cuticular Hb2 OS=Homo sapiens GN=KRT82 PE=1 SV=3
KRT83_HUMAN	70	55928	23	2	18	2	0.13	Keratin, type II cuticular Hb3 OS=Homo sapiens GN=KRT83 PE=1 SV=2
RL11_HUMAN	65	20468	11	3	9	3	0.66	60S ribosomal protein L11 OS=Homo sapiens GN=RPL11 PE=1 SV=2
H31T_HUMAN	58	15613	16	2	10	2	0.55	Histone H3.1t OS=Homo sapiens GN=HIST3H3 PE=1 SV=3
HNRPM_HUMAN	42	77749	60	2	38	2	0.1	Heterogeneous nuclear ribonucleoprotein M OS=Homo sapiens GN=HNRNPM PE=1 SV=3
PP2AA_HUMAN	35	36142	9	2	6	2	0.21	Serine/threonine-protein phosphatase 2A catalytic subunit alpha isoform OS=Homo sapiens GN=PPP2CA PE=1 SV=1
TITIN_HUMAN	24	3842904	758	2	569	2	0	Titin OS=Homo sapiens GN=TTN PE=1 SV=4
HNRPC_HUMAN	20	33707	18	2	13	2	0.23	Heterogeneous nuclear ribonucleoproteins C1/C2 OS=Homo sapiens GN=HNRNPC PE=1 SV=4
