# Supplementary material for: Proteomic and cellular localisation studies suggest non‐tight junction cytoplasmic and nuclear roles for occludin in astrocytes
Source: Eur J Neurosci. 2018 May 30;47(12):1444–56. doi: 10.1111/ejn.13933 (PMC6079634; doi:10.1111/ejn.13933)
Supplement: Supplementary file 1 [file EJN-47-1444-s001.pdf]

EJN European Journal of Neuroscience FENS Federation of European Neuroscience Societies

Proteomic and cellular localisation studies suggest non-tight junction cytoplasmic and nuclear roles for occludin in astrocytes

Ssarah Morgan, Claire Garwood, Luke Jennings, Julie Simpson, Lydia Castelli, Paul Heath, Simeon Mihaylov, Irina Vazquez Villasenor, Thomas Minshull, Paul Ince, Mark Dickman, Guillaume Hautbergue, Stephen Wharton

Review timeline:

Submission date:	26 June 2017
Editorial Decision:	2 August 2017
Revision received:	12 March 2018
Accepted:	16 April 2018

Editor: Masahiko Watanabe

1st Editorial Decision

Dear Dr. Garwood,

Your manuscript was reviewed by external reviewers as well as by the Section Editor, Prof. Masahiko Watanabe, and ourselves. Based on these reviews, we regret to inform you that we are not able to accept your manuscript for publication in EJN in its present form. However, the research described in your manuscript is potentially of interest and therefore we invite you to resubmit a much revised version.

The reviewers collectively indicated that your experiments generated new and important information. However, there are several critical issues that need to be resolved before we can further consider your manuscript for publication. Most importantly, both reviewers pointed out that the authors need to verify the specificity of antibodies against occludin. Specifically, Reviewer 1 considers that knockdown experiments should be performed to validate multiple bands obtained in the western blotting. Reviewer 2 also considers that you should confirm that procedures such as dephosphorylation protocol remove the extra bands. Accordingly, the revisions would need to resolve this and other important as well as minor issues.

Based on our own reading, we would like to point out that the authors need to improve presentation of immunohistochemical data (Figures 2 and 3) and explicitly show the subcellular localization and colocalization of two proteins. We also noted the following points.
- Referencing is not in EJN style
- Several abbreviations not defined at first use
- Dilutions needed for secondary antibodies used for western blots
- Details and dilution of biotinylated secondary antibody are not described.

Obviously, a resubmission of your manuscript could only be considered on the basis of extensive revisions, requiring the description of new evidence and/or additional analyses of your data. When you prepare your re-submission, please consult the Author's Checklist provided at the bottom on this email, notably with regard to characterization of reagents and reporting of statistical analyzes in the Results section and Figure legends.

Thank you for submitting your work to EJN.

Kind regards,

Paul Bolam & John Foxe

co-Editors in Chief, EJN

Reviews:

Reviewer: 1 Masanori Tachikawa (Tohoku University, Japan)

Comments to the Author
Sarah et al. have demonstrated the protein expression of occludin in the cytoplasm and nucleus of human cultured astrocytes and the binding proteins of occludin N-terminus and C-terminus polypeptides. Although the topic is potentially interesting, the experimental design and the basic data interpretation are still questionable. The present study also provides the unfocused results on the changes in occludin expression under neuropathological conditions. These considerations leave the impression of an uncompleted study. This reviewer raised the following points which should be addressed by the authors

Major comments
1. Figures 1 and 2: Immunoblots detected the multiple bands which were predicted to be the phosphorylated form, un-modification form, and degraded form of occludin by the authors. However, it appears that the sizes of detected bands are different in the whole cells lysates and/or cytoplasmic/nucleus fractions of 1321N1 cells and primary cultured astrocytes. Therefore, the specificity of the antibody (71-1500) is still questionable. The authors should provide the evidence showing the knockdown of occluding protein would result in the decreases in the intensities of the multiple bands.
2. Figure 1 and MS analysis: The recombinant proteins of occludin N- and C-terminus polypeptides and the whole cell lysate of 1321N1 cells were used for the pulldown assays. In Figure 1, only the anti-occludin antibody against the C-terminus polypeptide was used for immunoblotting analysis. The authors should present the data showing that the occludin proteins in the cytoplasmic and nucleus fractions would have the N-terminus sequences.
3. Figure 3: It would be better to provide the data showing that the immunoreactivities of occludin were detected in the cytoplasm and nucleus of astrocytes.
4. Supplemental Figure 2: How do the authors eliminate the possibility that the proteins, which were detected in MS analysis, could bind with the degraded form of the recombinant protein.
5. The data-dependent comprehensive MS analysis listed a large numbers of protein candidates with various reliabilities of the protein identifications. As the authors described in the Results section, the validation study using the immunoblot and/or knockdown techniques is necessary to discuss any functional significance of occludin-the detected proteins interactions.
6. Page 10 the last paragraph: It appears that this part of the discussion regarding the changes in occludin expression in the diseases would not be directly related to the conclusion.

Minor comments
1. Figure 2: Please add the A, B, and C in the Figure.
2. Supplemental Figure 1: What does "A." mean in the drawing ?
3. Page 8: Please indicate Figure 2A, 2B and Figure 3A, 3B in the text.

Reviewer: 2 Gareth John (Icahn School of Medicene at Mount Sinai, USA)

Comments to the Author
Occludin (OCLN) is a component of tight junctions, which are essential structural components of the blood brain barrier. OCLN is also expressed in cells without tight junctions, but its functions in these lineages remain unclear. In this manuscript, the authors have investigated the expression and localisation of occludin in astrocytes, microglial cells of the CNS, in cell culture and in human brain tissue. Additionally, they have used a proteomic approach to identify novel OCLN binding proteins in these cells.

The authors show using immunocytochemistry and immunohistochemistry that in the 1321N1 astrocytoma cell line, and in ScienCell human primary astrocytes, and also in human autopsy brain tissue, that OCLN is expressed in both astrocytic cytoplasm and nucleus. Additionally, using mass spectrometry, they identify binding of OCLN to nuclear and cytoplasmic proteins, particularly those related to RNA metabolism and nuclear function. On the basis of their data, they suggest that OCLN

may have novel functions in neuroepithelial cells that are unrelated to tight junction formation, and that these may be important in both physiological cell function and in disease states.

This is an interesting manuscript, and insightful, in that it addresses the functions of a tight junction-related protein in lineages that don't express tight junctions. The functions of OCLN (and MARVEL proteins more widely) are a bit of a conundrum, and so this work also has wide interest in cell biology more widely. If published, it is likely to be widely cited. Also, in general, I think the work in this paper is really well done, and many of the necessary controls have been included. Nonetheless, I do have some suggestions as to how the manuscript might be improved. These are optional, and the extent to which they are enforced are up to the editors. The suggestions are:

1. Abstract, final sentence. I would suggest that it might be better to write the second part of this sentence as a new sentence. Something akin to 'Further research will establish the roles of these functions in both cellular physiology and in disease states.'

2. Introduction. I think it might be a good idea to include a few sentences that contrast the phenotypes of Claudin knockouts with that of the Occludin knockout mouse. Much of this work was done by Shoichiro Tsukita's lab in Kyoto, and it really well illustrates the differences between the roles of claudins vs MARVEL proteins in tight junction strands. The authors could use these papers to illustrate their point that the functions of OCLN are not as simple as they first appear.

3. Results. Western blots and immunostaining. I know that the authors have worked with the best tools available. It might be a good idea to include data obtained using a different antibody for OCLN in these studies, if possible. Also (optional), if there are additional bands on Westerns that might be attributable to post-translational modification (phosphorylation), it might be a good idea to use a dephosphorylation protocol prior to gel loading to see if this removes the extra bands. Calf alk phos would be one way to do this, and commercial kits are available that are pretty inexpensive.

4. Pulldown/mass spec. This work is really interesting, and most of the controls have been done. One additional (optional) control would be to compare pulldown from the OCLN peptides, with that obtained using a scrambled peptide of the same length and similar hydrophilic/phobic content. Probably beyond the scope of this paper, but a thought for the future.

5. Pulldown/mass spec. This point seems important to me. The cohort of proteins identified by these elegant studies is not really discussed. The authors really should include gene ontology analysis of overall functional integration at minimum, to help the reader understand the potential functions of OCLN in cell lineages that don't make tight junctions. This can be done online and most institutions have a license for the relevant software. If the authors have identified a really interesting cohort of genes, why not explore the functional relevance of this information?

6. I would like to see a bit more depth regarding the statistical methods used in the mass spec analysis. A bit of mathematical detail in the methods will give this paper more weight.

Authors' Response 22 March 2018

Dear Prof. Bolam and Prof. Foxe,

Revision of manuscript EJN-2017-06-24710

Thank you for giving us the opportunity to revise our paper for your journal. We appreciate the constructive comments from yourselves and the referees, and hope that we have satisfactorily addressed the points raised. The changes made to our paper (highlighted in the manuscript) and our responses are as follows:

Response to Editors

• Referencing is not in EJN style.

EJN European Journal of Neuroscience FENS Federation of European Neuroscience Societies

Referencing has now been formatted correctly.

• Several abbreviations not defined at first use
Abbreviations are now defined.

• Dilutions needed for secondary antibodies used for western blots
These are now provided.

• Additional co-authors have been added to the author list. These co-authors all contributed to the revised manuscript. L Jennings contributed to additional cell culture work, LM Castelli assisted with cell fractionations and TC Minshull reanalysed the mass spectrometry to calculate the false discovery rate.

Response to Reviewer 1

• Figures 1 and 2: Immunoblots detected the multiple bands which were predicted to be the phosphorylated form, un-modification form, and degraded form of occludin by the authors. However, it appears that the sizes of detected bands are different in the whole cells lysates and/or cytoplasmic/nucleus fractions of 1321N1 cells and primary cultured astrocytes. Therefore, the specificity of the antibody (71-1500) is still questionable. The authors should provide the evidence showing the knockdown of occluding protein would result in the decreases in the intensities of the multiple bands.
We agree that the banding pattern for occludin differed between the human primary astrocytes, human astrocytoma line (1321N1) and the fractionated cells. This most likely reflected the fact that these western blots were performed at different times with different batches of the occludin polyclonal antibody (71-1500). The reviewer suggested knockdown of occludin to confirm specificity of the antibody which is beyond the scope of the current study, however we think we have satisfactorily addressed the concerns relating to antibody specificity. We have: 1) Harvested and assayed lysates from human primary astrocytes and 1321N1 cells simultaneously and run these lysates on the same gel. Membranes were probed with a new batch of the 71-1500 occludin antibody and the banding pattern is now similar, with a clear prominent band at 75kDa as well as additional bands which vary in size from ~35kDa - ~150kDa. 2) We have fractionated 1321N1 cells and probed with an additional occludin antibody directed against the N-terminus of occludin. 3) We pre-adsorbed the 71-500 occludin antibody with recombinant C-terminal occludin (aa 271-522) overnight before incubating with membranes and saw a clear reduction in the 75kDa, ~50kDa and 37kDa bands (supplementary figure 2).

• Figure 1 and MS analysis: The recombinant proteins of occludin N- and C-terminus polypeptides and the whole cell lysate of 1321N1 cells were used for the pulldown assays. In Figure 1, only the anti-occludin antibody against the C-terminus polypeptide was used for immunoblotting analysis. The authors should present the data showing that the occludin proteins in the cytoplasmic and nucleus fractions would have the N-terminus sequences.
We have now provided blots of fractionated 1321N1 astrocytoma cells probed with antibodes directed against the C-terminal (Thermofisher 71-1500) and the N-terminal (Abcam ab167161) . Both antibodies detect occludin in both cell fractions.

• Figure 3: It would be better to provide the data showing that the immunoreactivities of occludin were detected in the cytoplasm and nucleus of astrocytes.
We have included an additional image in this panel (image E) showing clear occludin nuclear reactivity. Image B clearly shows cytoplasmic occludin reactivity within an astrocyte. In addition the dual stained image (F) confirms localisation of occludin in both cellular compartments in GFAP-positive astrocytes.

• Supplemental Figure 2: How do the authors eliminate the possibility that the proteins, which were detected in MS analysis, could bind with the degraded form of the recombinant protein.
Now supplementary figure 3. We do not exclude the possibility that some of the proteins identified by MS interact with a truncated form of occludin however this does not affect the fact that the co-purified proteins still interact with occludin. This experiment is not designed to precisely define the protein binding-domains of occludin. We acknowledge the reviewer pointing out that the term "degraded" was likely not the best terminology to use and have now replace "degraded" by "truncated" forms of

occludin.

• The data-dependent comprehensive MS analysis listed a large numbers of protein candidates with various reliabilities of the protein identifications. As the authors described in the Results section, the validation study using the immunoblot and/or knockdown techniques is necessary to discuss any functional significance of occludin-the detected proteins interactions.
We agree that validation using immunoblot would be necessary to confirm the functional significance of these interactions. However, the primary purpose of the paper is to describe the expression of a tight junction protein in non-tight junction forming cells. The mass spectrometry was performed to identify potential interacting proteins other than those involved in tight formation and function (such as ZO-1).

• Page 10 the last paragraph: It appears that this part of the discussion regarding the changes in occludin expression in the diseases would not be directly related to the conclusion.
I'm unsure what the reviewer is referring to with this comment, we make no conclusion relating to disease in the final summary conclusion. The early points relating to disease highlight that these proteins are disrupted in astrocytes in disease which may provide further insights into additional roles of occludin.

• Figure 2: Please add the A, B, and C in the Figure. Supplemental Figure 1: What does "A." mean in the drawing ? Page 8: Please indicate Figure 2A, 2B and Figure 3A, 3B in the text.
These have all been amended/clarified.

Response to Reviewer 2

• Abstract, final sentence. I would suggest that it might be better to write the second part of this sentence as a new sentence. Something akin to 'Further research will establish the roles of these functions in both cellular physiology and in disease states.
We have amended this as suggested.

• Introduction. I think it might be a good idea to include a few sentences that contrast the phenotypes of Claudin knockouts with that of the Occludin knockout mouse. Much of this work was done by Shoichiro Tsukita's lab in Kyoto, and it really well illustrates the differences between the roles of claudins vs MARVEL proteins in tight junction strands. The authors could use these papers to illustrate their point that the functions of OCLN are not as simple as they first appear
We agree that the inclusion of some discussion relating to claudin-deficient versus occludin-deficient mice would enhance the introduction and have included an additional paragraph to cover this.

• Results. Western blots and immunostaining. I know that the authors have worked with the best tools available. It might be a good idea to include data obtained using a different antibody for OCLN in these studies, if possible. Also (optional), if there are additional bands on Westerns that might be attributable to post-translational modification (phosphorylation), it might be a good idea to use a dephosphorylation protocol prior to gel loading to see if this removes the extra bands. Calf alk phos would be one way to do this, and commercial kits are available that are pretty inexpensive.
We have used an additional antibody directed against the N-terminal of occludin as well as repeating blots with the original antibody (Thermofisher 71-1500, directed against the C-terminal). The C-terminal antibody recognised a clear band at 75kDa whilst the N-terminal detected a clear band at 65kDa (predicted size according to antibody manufacturer's). This difference is not unexpected given that the vast majority of occludin phosphorylation sites reside in the C-terminus.

• Pulldown/mass spec. This work is really interesting, and most of the controls have been done. One additional (optional) control would be to compare pulldown from the OCLN peptides, with that obtained using a scrambled peptide of the same length and similar hydrophilic/phobic content. Probably beyond the scope of this paper, but a thought for the future.
This is a very good suggestion but as the reviewer suggests, is beyond the scope of the current study.

• Pulldown/mass spec. This point seems important to me. The cohort of proteins identified by these elegant studies is not really discussed. The authors really should include gene ontology analysis of overall functional integration at minimum, to help the reader understand the potential functions of

OCLN in cell lineages that don't make tight junctions. This can be done online and most institutions have a license for the relevant software. If the authors have identified a really interesting cohort of genes, why not explore the functional relevance of this information?
This analysis was originally performed using the Search Tool for the Retrieval of Interacting Genes/Proteins (STRING) but none of the proteins identified by the mass spectrometry experiments were identified in the gene ontology analysis. This has since been revisited; one protein identified in the MS analysis was also identified using STRING (ELAV1L). We think this most likely reflects the fact that, to date, work on occludin has almost exclusively been performed in relation to tight junctions (to highlight this 25 of the top 50 hits in the STRING analysis were claudins) with little consideration of potential functions outside of this role.

• I would like to see a bit more depth regarding the statistical methods used in the mass spec analysis. A bit of mathematical detail in the methods will give this paper more weight.

We have provided additional details to reflect the criteria used for protein identifications (see page 8). A peptide ion score of ≤ 15 as a cut-off as calculated by Mascot was also used to filter. Proteins were identified using a significance threshold $p < 0.05$. Protein identifications were based on a minimum of two unique peptides and a Mascot score ≥ 50 were considered to be significant. The false discovery rates were determined using the Mascot decoy database option and were typically $\leq 3\%$.
